# One, two or three? Probing the stoichiometry of membrane proteins by single-molecule localization microscopy

**DOI:** 10.1038/srep14072

**Published:** 2015-09-11

**Authors:** Franziska Fricke, Joel Beaudouin, Roland Eils, Mike Heilemann

**Affiliations:** 1 Institute of Physical and Theoretical Chemistry, Goethe-University Frankfurt, Max-von-Laue-Str. 7, 60438 Frankfurt am Main, Germany; 2 Department for Bioinformatics and Functional Genomics, Bioquant and Institute of Pharmacy and Molecular Biotechnology, University of Heidelberg, and Division of Theoretical Bioinformatics, German Cancer Research Center (DKFZ), Im Neuenheimer Feld 267, 69120 Heidelberg, Germany

## Abstract

Probing the oligomeric state of abundant molecules, such as membrane proteins in intact cells, is essential, but has not been straightforward. We address this challenge with a simple counting strategy that is capable of reporting the oligomeric state of dense, membrane-bound protein complexes. It is based on single-molecule localization microscopy to super-resolve protein structures in intact cells and basic quantitative evaluation. We validate our method with membrane-bound monomeric CD86 and dimeric cytotoxic T-lymphocyte-associated protein as model proteins and confirm their oligomeric states. We further detect oligomerization of CD80 and vesicular stomatitis virus glycoprotein and propose coexistence of monomers and dimers for CD80 and trimeric assembly of the viral protein at the cell membrane. This approach should prove valuable for researchers striving for reliable molecular counting in cells.

In the membrane sciences, protein stoichiometry is often equated with cellular function. Many cell-surface proteins such as ion channels, transporters and receptors are suspected of forming oligomers or even changing their oligomeric state in order to fulfill a certain task. Several membrane receptors including G protein-coupled receptors^1^, cytokine and growth factor receptors^2^,^3^ have been proposed to oligomerize upon ligand binding, presumably a prerequisite for intracellular signal initiation. Advances in single-molecule fluorescence imaging^4^ have brought molecular counting within the native membrane environment in direct reach. For example, single-molecule microscopic techniques indicate a preexistence and functional role of dimers prior to ligand activation for many receptor tyrosine kinases^5^,^6^,^7^,^8^,^9^,^10^ among others. However, rigorous single-molecule studies of membrane protein organization and stoichiometry on intact cells are rare owing to technical challenges and methodological limitations. For instance, single-molecule photobleaching^11^, a solid, but diffraction-limited method for subunit counting, is restricted to low expression levels of less than 1–2 protein complexes per μm^2^ at the cell membrane^12^.

Recent developments and applications have demonstrated the potential of single-molecule localization microscopy (SMLM) for studying the organization of membrane proteins in intact cells. SMLM summarizes several variants, such as photoactivated localization microscopy (PALM)^13^, fluorescence photoactivation localization microscopy (FPALM)^14^, stochastic optical reconstruction microscopy (STORM)^15^, *direct* STORM (*d*STORM)^16^, and ground state depletion microscopy followed by individual molecule return (GSDIM)^17^, all capable of circumventing the optical diffraction limit to visualize structures at the nanoscale. They rely on bright photoswitchable fluorescent probes, namely fluorescent proteins and organic dyes, that are activated in sparse numbers over time and localized with high precision. Coordinates of individual fluorophores construct the super-resolved image but can also be exploited for quantitative evaluation including spatial distribution analysis and molecular counting. In contrast to other single-molecule methods, SMLM is not limited to low protein surface densities owing to its intrinsic capability for super-resolution imaging. Fluorescent proteins (FPs) are particularly attractive probes for quantitative SMLM because they allow stoichiometric labeling of the target structure and do not require dye incubation of cells prone to unspecific staining.

Reliable protein quantification using SMLM is, however, hampered: The blinking behavior of photoswitchable probes can introduce overcounting artifacts^18^. This can be overcome by introducing an empirical global dark time that combines fluorophore emissions closely clustered in time^18^,^19^,^20^,^21^,^22^. Undercounting can arise when not every target is fluorescently tagged or blinking cycles of separate fluorophores overlap in time caused by high protein densities, fast photoactivation schemes or extended blinking of the fluorescent probe.

Today, there are few strategies that truly overcome the technical difficulties of molecular counting with SMLM. The pair-correlation approach^23^,^24^ is useful for analyzing structures that are larger than the effective resolution of the measurement. By comparing the pair-correlation function to a model, various parameters including cluster size and the number of proteins per cluster can be derived. The method developed by Lee and co-workers^25^ corrects for overcounting of FPs, but requires detailed knowledge of photokinetic rates to estimate the optimal global dark time. A recent improvement^26^ based on an aggregated Markov model is independent of a temporal threshold and *a priori* knowledge of photokinetic parameters. The method is sophisticated, but computationally advanced, and, so far, mostly synthetic data sets have been analyzed. Moreover, it is rarely the case that all photoswitchable probes are photodetectable^27^, though frequently ignored in the past. Undetectable fractions of fluorescent proteins are largely attributed to incomplete maturation, misfolding, protonation states or premature photobleaching of the fluorescent probe^11^,^28^,^29^. There were several attempts to determine the fraction of photodetectable FPs, but results are controversial and could depend on expression systems among other factors. For example, the photodetectable fraction of photoactivatable PAmCherry FP was found to be 4% in *E. coli*^30^, 45% in *Xenopus* oocytes^27^, and 77% in BHK21 cells^21^.

Here, we present a simple strategy for extracting the stoichiometry of membrane proteins from SMLM data. Our method directly relates the number of fluorophore localizations to the number of underlying molecules, and takes into account the stochastic nature of FP blinking to overcome impediments of dark time thresholding. We validated our strategy with monomeric and dimeric proteins as standards and revealed the oligomeric states of membrane proteins in intact cells. Our findings demonstrate reliable molecular counting and the capability to distinguish between monomers, dimers and higher-order oligomers. Finally, our counting strategy is intuitive and easily implemented and puts quantification of small protein complexes in immediate reach of interested users.

## Results

### Molecular Counting Strategy

**Figure 1:**
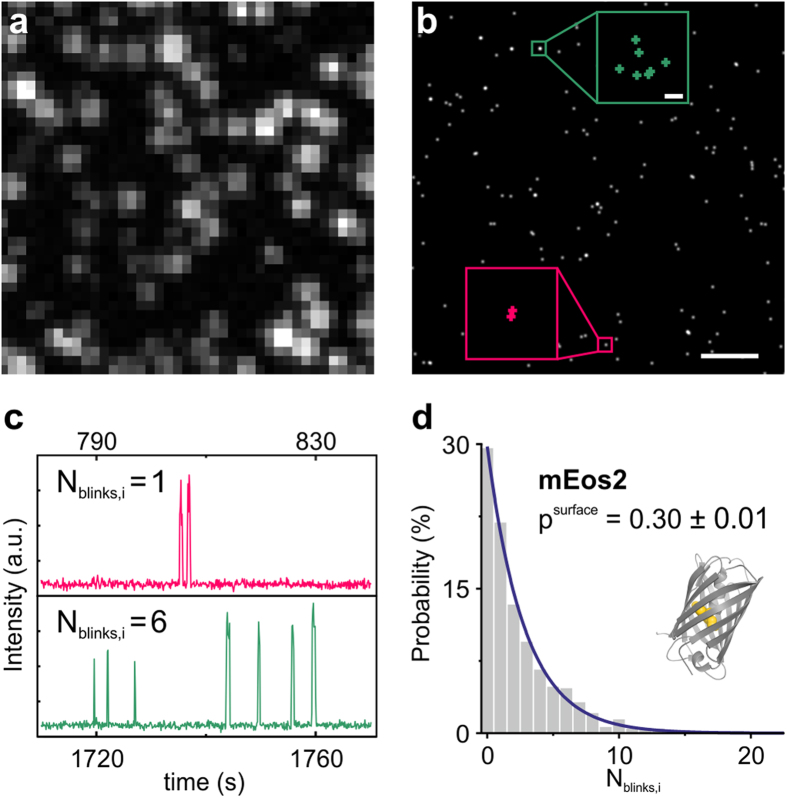
Single mEos2 blinking characteristics. TIRF (**a**) and SMLM (**b**) image of mEos2 single-molecule surface. The boxed insets in (**b**) are magnifications of two mEos2 molecules and demonstrate repeated localizations (crosses) of single mEos2 FPs. (**c**) Intensity time traces of mEos2 molecules boxed in (**b**) show distinct blinking after photoactivation before the FPs photobleach irreversibly. Time traces (**c**) and crosses (**b**) having the same color belong to the same molecule. (**d**) Distribution of the number of blinking events N_blinks,i_ upon photoactivation for single mEos2 FPs. The distribution is fit to a geometric distribution with p^surface ^= 0.30 ± 0.01 as fitting parameter (n = 622 mEos2 molecules, adjusted R^2 ^= 0.995) Note that N_blinks,i_ corresponds to the number of fluorophore reactivations, not total appearances. Scale bars: 1 μm; inset 10 nm.

Previous studies have shown that the photokinetics of many photoswitchable FPs used for SMLM are well described by a simple four-states model^20^,^25^ (Supplementary Fig. S1): Once activated, these probes can switch between a non-fluorescent and a fluorescent state, commonly referred to as FP blinking, before irreversible photobleaching occurs. The model predicts that the distribution of the number of times a single FP (i) blinks upon photoactivation (N_blinks,i_) resembles a geometric distribution 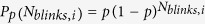  25. Here, p is the probability to observe no blinking of the fluorescent probe. We verified this model using single-molecule surfaces of bacterially expressed and purified mEos2 (Fig. 1a,b). Upon imaging under SMLM conditions, intensity time traces of single mEos2 molecules were extracted (Fig. 1c) to count the number of blinking events per mEos2 protein. This generates a distribution of N_blinks,i_ well approximated by a geometric distribution with p^surface ^= 0.30 ± 0.01 with confidence interval (Fig. 1d). We therefore assume that the kinetic model is valid and suitable for describing the blinking statistics of mEos2 FP. Please note, that the blinking parameter p is not a constant inherent to the respective FP. FP photophysics are dependent on environmental factors, such as buffer conditions and illumination density for fluorophore excitation/switching^18^,^19^,^31^. This is nicely demonstrated by the different probabilities of mEos2 not blinking obtained by Lee *et al.* (p = 0.41)^25^ and us.

In case of oligomerization, the number of emitting molecules N is larger than one. However, the FPs exhibit identical blinking kinetics summarized by the blink parameter p, given that all FPs behave similarly. The distribution of 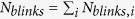 then follows a negative binomial distribution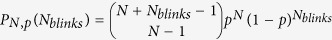.This distribution is merely a generalization of the geometric distribution (the latter is readily obtained for N = 1). Our strategy goes as follows (Supplementary Fig. S1): Single protein complexes are determined from SMLM data sets to extract the number of blinking events per complex, which corresponds to N_blinks_. The distribution of N_blinks_ is then fitted to a negative binomial distribution yielding the number of underlying molecules N as free fit parameter.

### Validation of molecular counting

**Figure 2:**
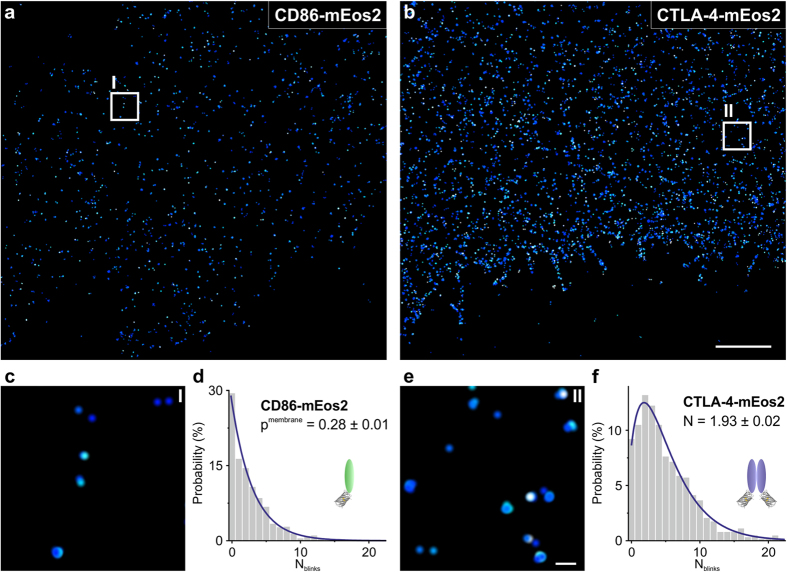
Validation of SMLM counting strategy using monomeric and dimeric model proteins. SMLM images of CD86-mEos2 (**a**) and CTLA-4-mEos2 (**b**) at the membrane of Hela cells. (**c**,**e**) Magnifications of region I or II boxed in (**a**) or (**b**) respectively. The inset shows a magnification of the boxed region. Distributions of N_blinks_ are generated from SMLM data sets. (**d**) For CD86-mEos2, the distribution is well approximated by a geometric distribution and yields p^membrane ^= 0.28 ± 0.01 as blink parameter, which is used as a fixed value for all other membrane proteins (n = 11 cells, adjusted R^2 ^= 0.986 for CD86-mEos2). (**f**) For CTLA-4-mEos2, the distribution of N_blinks_ fits to a negative binomial distribution with an average number of N = 1.93 ± 0.02 underlying fluorescent probes per complex (n = 11 cells, adjusted R^2 ^= 0.989 for CTLA-4-mEos2). Scale bars: 2 μm; inset 100 nm.

We tested our counting strategy using proteins of well-known and defined stoichiometry tagged with mEos2 FP. We chose two membrane receptors: monomeric receptor CD86 and covalent dimeric cytotoxic T-lymphocyte-associated protein 4 (CTLA-4). These proteins show little or no endogenous expression in Hela cells and their respective oligomeric state is well described and validated by various studies in the past (see Supplementary Notes). Both receptors were fused with mEos2 on the intracellular side and expressed in Hela cells. SMLM images were acquired of fixed cells with moderate expression levels at the cell membrane ranging from 3–24 protein complexes per μm^2^ for CD86-mEos2 and 2–25 protein complexes per μm^2^ for CTLA-4-mEos2 (Supplementary Fig. S2). SMLM images demonstrate membrane localization for both proteins, as expected (Fig. 2a–c,e). Using these data sets, we generated N_blinks_ distributions of spatially clustered mEos2 (Fig. 2d,f). For CD86-mEos2, the distribution of blinking events is well described by a geometric distribution with blink parameter p^membrane ^= 0.28 ± 0.01 (Fig. 2d), almost identical to p^surface ^= 0.30 ± 0.01 obtained for single mEos2 surfaces. Similar results were obtained when fitting with a negative binomial distribution (Supplementary Fig. S3). Our findings verify the monomeric assembly of CD86-mEos2 and suggest that different expression systems and the membrane microenvironment have negligible effects on the blinking statistics of mEos2 fluorescent proteins under our imaging conditions. We decided to use p^membrane^ as a fixed fit parameter for all other proteins, as it takes account of FP blinking statistics at the membrane and reduces the number of fitting parameters to one.

Next, we probed the stoichiometry of CTLA-4-mEos2. The distribution of N_blinks_ was extracted and fitted with a negative binomial distribution to obtain the number of underlying molecules per mEos2 cluster. We obtained N = 1.93 ± 0.02 as fitting parameter indicating a dimeric assembly of CTLA-4-mEos2 (Fig. 2f). To verify whether it is valid to use p^membrane ^= 0.28 for other membrane proteins, a similar fit was employed where the blink parameter p is a fitting parameter (Supplementary Fig. S4). The obtained value p = 0.27 ± 0.01 is in good agreement with the blink parameter p^membrane^ of CD86-mEos2. This suggests, that the blinking behavior of mEos2 is little affected by the fusion membrane protein. A comparison with two models that directly account for trimers verifies that, first, a model based on an average number of molecules N is sufficient to describe the data set and, second, it is unlikely that CTLA-4-mEos2 forms higher-order oligomers (Supplementary Fig. S5). As expected, the oligomerization N is smaller than 2, because not every mEos2 FP is photodetectable. Since the constitutive dimerization of CTLA-4 at the cell membrane is well-known, N is used to estimate the probability for mEos2 photodetection. Approximately 90% of mEos2 molecules are detected under our experimental conditions (see Supplementary Methods). In summary, our molecular counting strategy readily identifies homomeric interactions of membrane proteins and reproduced predicted stoichiometries of CD86 and CTLA-4.

### Oligomeric assembly of CD80 and VSVG

**Figure 3:**
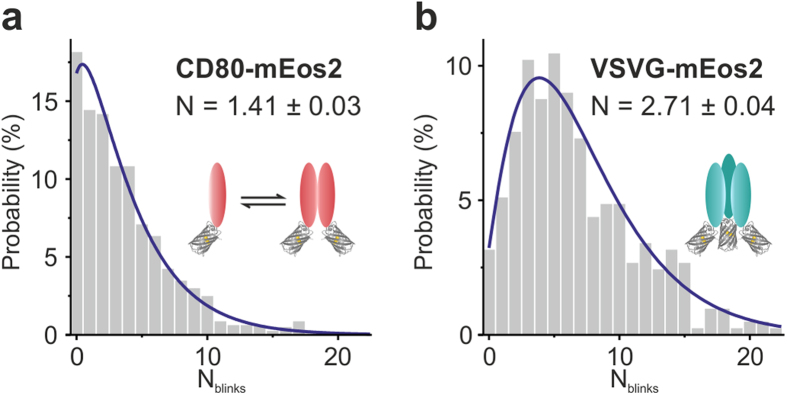
Probing the oligomeric states of membrane proteins with SMLM. VSVG-mEos2 and CD80-mEos2 were expressed in Hela cells and subjected to SMLM imaging. (**a**,**b**) Resulting distributions of N_blinks_ were fit to negative binomial distributions yielding N = 1.41 ± 0.03 for CD80-mEos2 (**a**) and N = 2.71 ± 0.04 for VSVG-mEos2 (**b**) as fitting parameters (n = 10 cells, adjusted R^2 ^= 0.983 for CD80-mEos2; n = 11 cells, adjusted R^2 ^= 0.964 for VSVG-mEos2).

Finally, we applied this approach to detect oligomerization of membrane-bound CD80 and vesicular stomatitis virus glycoprotein (VSVG). Both proteins were tagged with mEos2 at the intracellular side and expressed in Hela cells for SMLM analysis. Endogenous expression of CD80 was not observed in Hela cells (see Supplementary Notes) and the viral protein VSVG is not expressed in Hela cells. We imaged Hela cells exhibiting moderate protein levels at the membrane in the range of 1.5–10 protein complexes per μm^2^ for CD80-mEos2 and 0.5–7 protein complexes per μm^2^ for VSVG-mEos2 (Supplementary Fig. S2). For CD80-mEos2, the number of mEos2 molecules per complex (N = 1.41 ± 0.03) is lower than that obtained for CTLA-4 (Fig. 3a), indicating that CD80 monomers and dimers coexist at the plasma membrane. This interpretation is consistent with previous studies, which reported a dimeric self-association of CD80 *in vitro*^32^,^33^ and *in vivo*^34^,^35^, but unknown to what extent. Taking into account the mEos2 photodetection probability, we obtain an average oligomerization of 1.57 ± 0.03, which is equivalent to a dimeric fraction of (57 ± 3)% and a monomeric fraction of (43 ± 3)% of CTLA-4-mEos2. To further validate this finding, we evaluated a fitting model that explicitly includes a monomeric and a dimeric contribution (Supplementary Fig. S6) and obtain similar values for the dimeric/monomeric fraction. To rule out the possibility that CD80 forms trimers on the membrane, we tested a different model that included trimers. Fitting the experimental data with this model did not yield any trimeric contribution (Supplementary Fig. S6). Our results thus indicate that CD80 is found both monomeric and dimeric.

The blinking distribution of VSVG-mEos2 features a shift to higher values of N_blinks_ manifesting in a fitting parameter of N = 2.71 ± 0.04 (Fig. 3b). Considering the mEos2 photodetectability, we predict 3.01 ± 0.04 underlying molecules per complex which corresponds to a trimeric stoichiometry of VSVG at the cell membrane. This is in accordance with earlier studies that reported a trimeric organization of the virus protein as functional state^36^,^37^,^38^. For further validation, we evaluated contributions from trimers, dimers and monomers explicitly using a weighted sum of negative binomial distributions (Supplementary Fig. S7). Note, that a trimeric protein will appear dimeric/monomeric with a certain probability, when not every FP is detected (see Supplementary Methods). By comparing the obtained weights to the expected probabilities, we verify the trimeric assembly of VSVG-mEos2. We further applied a model function that included tetramers, but obtained no tetrameric contribution from the fit (see Supplementary Fig. S7).

## Discussion

Molecular counting with SMLM is promising, but remained a challenge for various reasons. We showed that the counting strategy presented here reliably predicts the oligomeric state of membrane proteins and is capable of distinguishing between monomers, dimers and oligomers of higher order. We confirmed the truly monomeric state of CD86 and stable dimerization of CTLA-4 at the membrane of intact cells. For CD80, we observed considerable levels of dimeric self-association, indicating an equilibrium of monomers and dimers. Our finding supports previous evidence of a dimeric pre-assembly of the protein, which is presumably enforced upon binding of its bivalent receptors CD28/CTLA-4 to form chain-like structures on the cell surface^39^. VSVG is organized in higher oligomers at the plasma membrane that we identified as trimers. This is consistent with early biochemical studies that reported trimer formation to be critical for transport of the viral glycoprotein to the cell membrane^36^,^40^.

Our molecular counting strategy is useful for many users because it is simple, intuitive and easily implemented. For this strategy, no advance knowledge of fluorophore photokinetics is necessary, the challenge of molecular counting is reduced to one-parameter curve fitting. One limitation of the strategy is the requirement of stoichiometric controls, e.g. monomers and dimers. These are useful for determining the photodetectable fraction and the blinking probability of the fluorescent probe for reliable protein quantification. For membrane proteins, we suggest CD86 and CTLA-4 to be employed as stoichiometric standards.

A key advantage of the proposed analysis is its versatility: It can be combined with almost any localization software capable of grouping fluorescence bursts in consecutive camera frames (e.g. RapidSTORM^41^, ThunderSTORM^42^, GraspJ^43^; all of them freely available) and with different spatial segmentation techniques for grouping localizations into clusters. The strategy is designed for probing the stoichiometry of membrane proteins, but can in principal be extended to other biological systems, where access to the oligomeric state is desired. Quantification of proteins with well-defined stoichiometry as well as mixed populations, such as monomers/dimers, or changes in oligomerization should all be accessible. It should prove particularly useful for membrane proteins that are suspected to oligomerize for certain tasks. Probing receptor stoichiometries in the absence and presence of ligands or co-receptors could link oligomeric states to cellular function such as signal transduction.

## Methods

### Plasmids and cell culture

The coding sequences of human CD80, CD86 and CTLA-4 were synthetically designed (Eurofins). The last 23 amino acids of CTLA-4 were removed by PCR to reduce the internalization of the receptor and concentrate it at the plasma membrane^44^. The three constructs were fused to mEos2 through a linker coding for GGGGGPVPQWEGFAALLATPVGGAV and cloned into the IRES-puro2 vector (Clontech). The pPAGFP-VSVG (ts045 mutant) plasmid and the pRSetA-mEos2 vector were obtained from Addgene (Addgene plasmids # 11915 and # 20341)^45^,^46^. To produce VSVG-mEos2 plasmid, mEos2 cDNA was amplified from mEos2-H2B plasmid^47^ from our lab using forward primer 5´-GCGCGCGGATCCAATGAGTGCGA-3´ and reverse primer 5´-GCGCGCTGTACATTATCGTCTGGCA-3´. VSVG-mEOS2 plasmid was generated by replacing PAGFP in pPAGFP-VSVG with mEos2 using BamH1 and Bsp14071 restriction sites.

Hela cells were maintained at 37 °C and in a 5% CO_2_ humidified atmosphere in DMEM (Life Technologies) supplemented with 10% fetal bovine serum (Biochrom or Life Technologies), 2 mM L-Glutamin or 1% GlutaMAX (both Life Technologies) and Penicillin-Streptomycin (Life Technologies).

### Microscopic sample preparation

For single-molecule mEos2 surfaces, glass slides (PLANO GmbH) were washed in isopropyl alcohol (Sigma) for 30 min, plasma cleaned in N_2_ plasma for 10 min (Diener Electronic) and incubated in 100 μg/ml poly-L-lysine solution (Sigma) for 2 h at room temperature. After washing in ddH_2_O, slides were dried under a laminar flow hood. mEos2 fluorescent protein was purified as described in detail elsewhere^48^. Single mEos2 surfaces where obtained by incubating the coated glass slides with 625 pM mEos2 protein solution in PBS (Sigma) for 30 min followed by repeated washing with PBS.

Microscopic samples with Hela cells expressing CD80-, CD86- and CTLA-4-mEos2 were prepared as follows: Hela cells were maintained in supplemented culture medium without phenol-red. Hela cells plated on plastic dishes were transfected with small amounts of plasmid diluted in salmon sperm DNA (Life Technologies) with X-tremeGENE HP (Roche). Three days later, an 8-well Labtek chambered coverglass was coated for one hour with 50 μM bovine fibronectin (Sigma). Cells were trypsinated and replated on the coverglass for three hours before fixation.

For VSVG-mEos2 imaging, glass slides were coated with a polyethylene glycol (PEG) brush (Rapp Polymere) to reduce background. The PEG was functionalized with poly-L-lysine (Sigma) on one end and an RGD peptide on the other to ensure cell attachment via RGD binding (please see VandeVondele *et al.*^49^ for more details). Glass slides were plasma cleaned as described above, then incubated in 0.8 mg/ml functionalized PEG for 2 h. After rinsing in ddH_2_O, slides were blow dried with N_2_ and could be stored at −20 °C for several weeks. Hela cells were seeded on PEG-coated glass slides and transfected with VSVG-mEos2 plasmid using Fugene HD (Promega). After 24 h, cells expressing VSVG-mEos2 were placed in an incubator at 32 °C overnight to ensure VSVG localization at the cell membrane before fixation.

All transfected Hela cells were fixed with a mixture of 4% formaldehyde (16% methanol-free stock from Thermo Scientific), 0.1% glutaraldehyde (25% stock from Sigma) and 0.4 M sucrose (Sigma) in PBS for 15–20 min followed by repeated washing with 0.4 M sucrose in PBS. Microscopic samples were imaged in PBS.

### Flow Cytometry

CD80, CD86 and CTLA-4 expression at the plasma membrane was measured by immunofluorescence on living Hela cells using PE tagged antibodies. The antibody against CD80 was the clone MEM-233 (Life Technologies), the one against CD86 was the clone IT2.2 (eBioscience) and the one against CTLA-4 was the clone 14D3 (eBioscience). Cells were trypsinated, blocked on ice with 1% bovine serum albumin, incubated for 30 min with antibody in blocking buffer on ice and directly measured by flow cytometry (Beckman Coulter).

### Single-molecule localization microscopy (SMLM)

SMLM was carried out on a custom-build laser microscope operated in total internal reflection fluorescence (TIRF) mode^50^. All lasers were coupled into an inverted microscope (Olympus IX71) equipped with a 100× oil immersion objective (PLAPO 100× TIRFM, NA ≥ 1.45, Olympus) via dichroic mirrors (AHF). mEos2 in its native form was excited with a 488 nm laser (Sapphire 488 LP, Coherent). For PALM, mEos2 was simultaneously photoconverted, imaged and photobleached by gradually increasing the UV illumination (405 nm; 0–10 W/cm^2^; CUBE 405-50C, Coherent) combined with continuous excitation at 568 nm (0.5 kW/cm^2^; Sapphire 568 LP, Coherent). Single-molecule fluorescence movies of mEos2 were recorded with a frame rate of 10 Hz using an EMCCD camera (iXon3, Andor) after bandpass filtering (BrightLine HC 590/20, AHF).

### SMLM data analysis

SMLM movies were analyzed using the rapidSTORM^41^ software and post-processed using a python-based custom written software. In brief, single mEos2 fluorescent peaks were identified in each frame and fit by a two-dimensional Gaussian function to extract spatial coordinates and the number of photons. Peaks with brightness below 63 photons were discarded. The majority of peaks were localized with a spatial precision <35 nm according to Mortensen *et al.*^51^ PALM images were generated from the coordinate lists of localized peaks using rapidSTORM or ViSP^52^. Spatio-temporal grouping was applied so that peaks appearing in consecutive camera frames within a spatial separation of 90 nm were registered as a single localization with averaged spatial position. The algorithm is based on Kalman filtering and is implemented in rapidSTORM. The distance threshold was chosen so that 99% of peaks with a localization precision of 35 nm were grouped successfully. Peaks that only appeared in one frame were excluded. Note that the temporal cutoff time does not correct for the long-lived fluorescent off times previously described for mEos2 that can last up to several seconds^18^. Individual molecules in a small protein complex (e.g. receptor dimer) cannot be spatially resolved with PALM and will appear as clusters of several localizations with a spatial spread in the range of PALM resolution itself. Before spatio-temporal grouping, even a monomeric protein will give rise to a cluster of localizations due to repeated mEos2 emissions. We made use of the characteristic blinking behavior of mEos2 and defined protein complexes as mEos2 bursts with a maximum spread of 100 nm in the initial PALM image. We rigorously excluded clusters with low brightness, partially overlapping clusters and clusters in close proximity to localizations caused by fluorescent background. mEos2 localizations identified as belonging to the same complex are then used to extract the true number of mEos2 reappearances/blinks (N_blinks_) per cluster after spatio-temporal grouping. Distributions of N_blinks_ were plotted, fitted and statistically evaluated using OriginPro 9.1G.

## Additional Information

**How to cite this article**: Fricke, F. *et al.* One, two or three? Probing the stoichiometry of membrane proteins by single-molecule localization microscopy. *Sci. Rep.* **5**, 14072; doi: 10.1038/srep14072 (2015).

## Supplementary Material

Supplementary Information
