# Supplementary material for: One, two or three? Probing the stoichiometry of membrane proteins by single-molecule localization microscopy
Source: Sci Rep. 2015 Sep 11;5:14072. doi: 10.1038/srep14072 (PMC4642553; doi:10.1038/srep14072)
Supplement: Supplementary Information [file srep14072-s1.pdf]

Supplementary information

One, two or three? Probing the stoichiometry of membrane proteins by single-molecule localization microscopy

Franziska Fricke[1,3], Joel Beaudouin[2,3], Roland Eils[2] and Mike Heilemann[1,*]

[1]Institute of Physical and Theoretical Chemistry, Goethe-University Frankfurt,
Max-von-Laue-Str. 7, Frankfurt am Main 60438, Germany

[2]Department for Bioinformatics and Functional Genomics, Bioquant and Institute of Pharmacy and Molecular Biotechnology, University of Heidelberg,
and Division of Theoretical Bioinformatics, German Cancer Research Center (DKFZ),
Im Neuenheimer Feld 267, Heidelberg 69120, Germany

[3]These authors contributed equally to this work

[*]Corresponding author

Supplementary Figure S1: Schematic illustration of molecular counting strategy using SMLM.

Supplementary Figure S2: Area densities of membrane protein complexes.

Supplementary Figure S3: Comparison of fitting models for CD86-mEos2.

Supplementary Figure S4: Evaluation of the blink parameter p for CTLA-4-mEos2.

Supplementary Figure S5: Comparison of fitting models for CTLA-4-mEos2.

Supplementary Figure S6: Comparison of fitting models for CD80-mEos2.

Supplementary Figure S7: Comparison of fitting models for VSVG-mEos2.

Supplementary Notes.

Supplementary Methods.

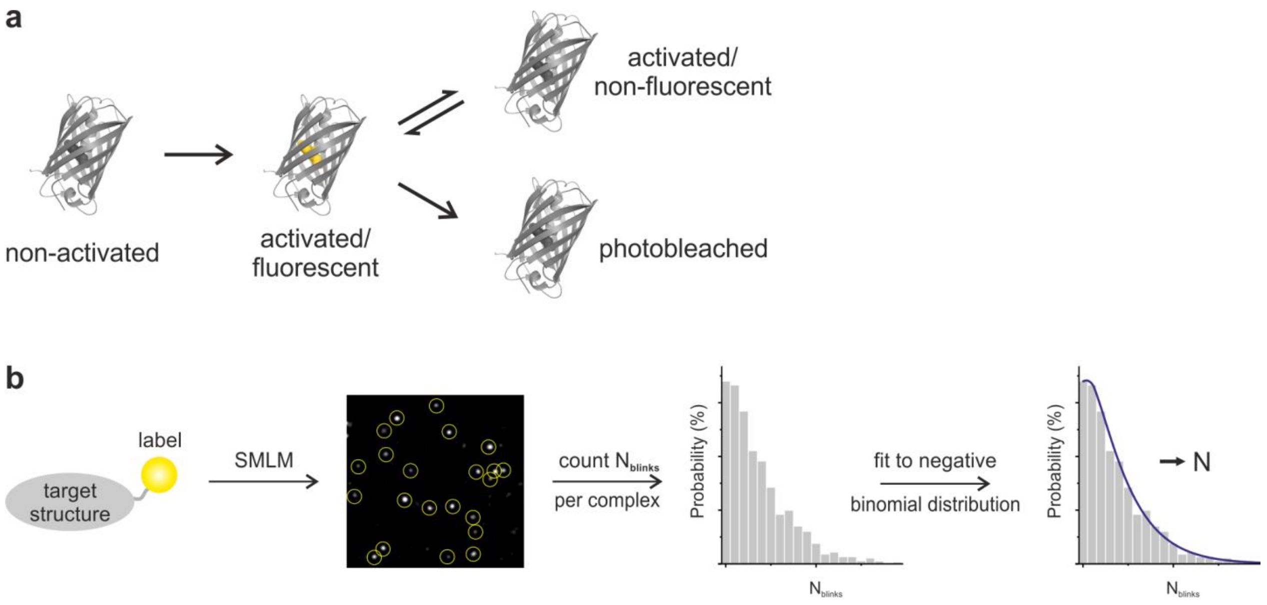

Supplementary Figure S1: (**a**) Photokinetic model for photoswitchable fluorescent proteins. (**b**) Schematic illustration of molecular counting strategy using SMLM. Cells expressing the target structure tagged with a fluorescent probe are imaged using SMLM. Spatially clustered complexes are identified to extract the number of blinking events N_{blinks} per complex. This generates a N_{blinks} distribution that is fitted with a negative binomial distribution yielding the number of detected molecules N.

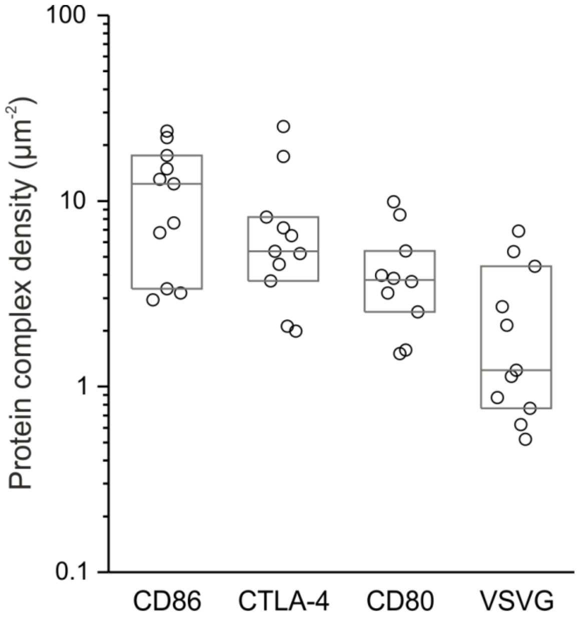

Supplementary Figure S2: Area density of CD86-mEos2 (n = 11 cells), CTLA-4-mEos2 (n = 11 cells), VSVG-mEos2 (n = 11 cells) and CD80-mEos2 (n = 10 cells) complexes at the cell membrane determined with SMLM. Box plots visualize the median value (line) and the 25th and 75th percentiles (box) for each data set.

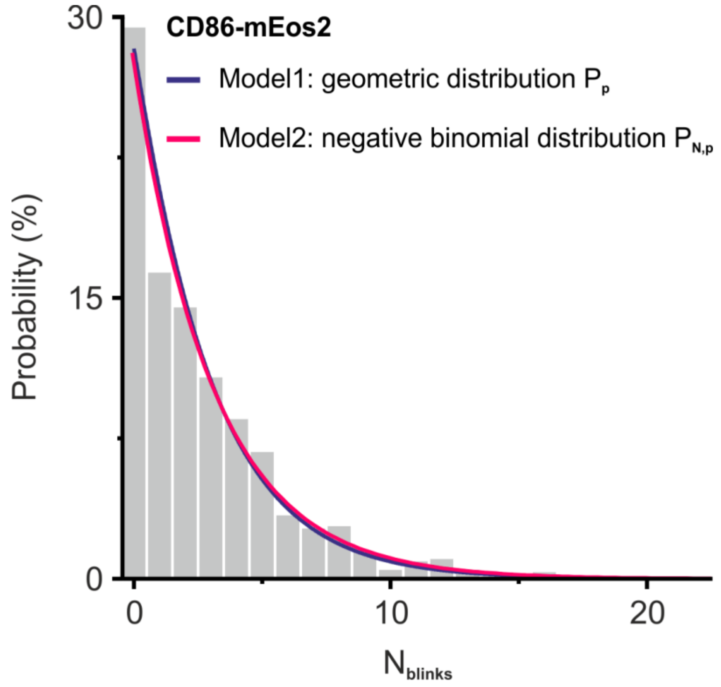

Supplementary Figure S3: Comparison of fitting models for CD86-mEos2. The probability distribution of N_{blinks} is well fit by a geometric distribution (Model1, blue, adjusted $R^2 = 0.986$), as expected for monomeric assembly. This is confirmed by fitting with the negative binomial distribution (Model2, magenta, adjusted $R^2 = 0.975$), where $N = 1.04 \pm 0.03$ is obtained as fitting parameter. The geometric distribution is 5 times more likely to be the correct fitting model compared to the negative binomial distribution based on Akaike's information criterion (AIC) test (AIC = -148.3 for geometric distribution; AIC = -144.1 for negative binomial distribution).

a

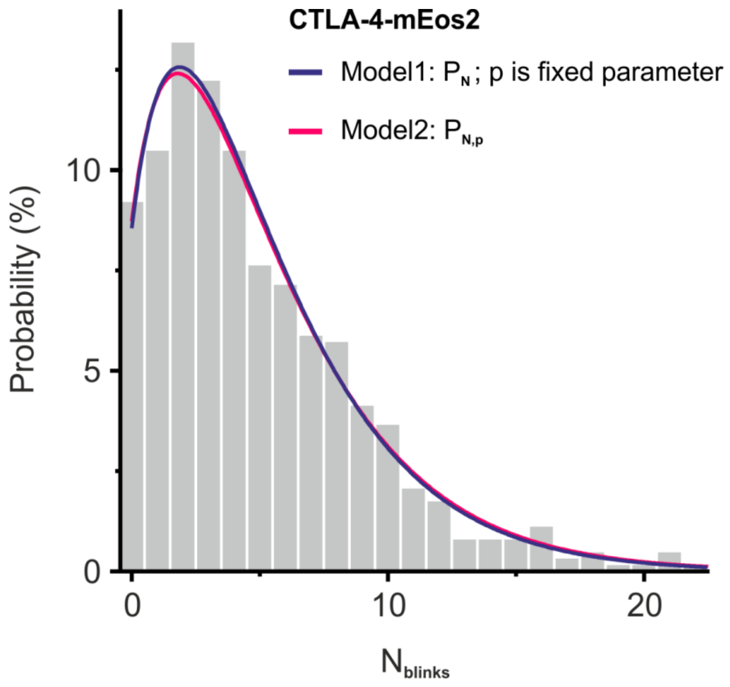

b

Fitting parameter	*Model1*	*Model2*
N	1.93 ± 0.02	1.86 ± 0.07
p	NA	0.27 ± 0.01
adjusted R^2	0.989	0.989
AIC	-576.7	-575.8

Supplementary Figure S4: Evaluation of the blink parameter p for CTLA-4-mEos2. (**a**) The distribution of N_{blinks} is fitted to two different models denoted by Model1 (blue) and Model2 (magenta). Both models are negative binomial distributions with N as fitting parameter. For Model1, the blink parameter of 0.28 was employed as fixed parameter, while p is set as free fitting parameter for Model2. (**b**) The respective fitting parameters, adjusted R^2 and AIC values are listed for all models. Model2 yields p = 0.27 ± 0.01 as fitting parameter, which agrees well with $p^{membrane}$ = 0.28 ± 0.01 as obtained for CD86-mEos2. We therefore conclude that the blinking behavior of mEos2 FP on the intracellular, membrane-proximal side is not majorly affected by the membrane fusion protein.

a

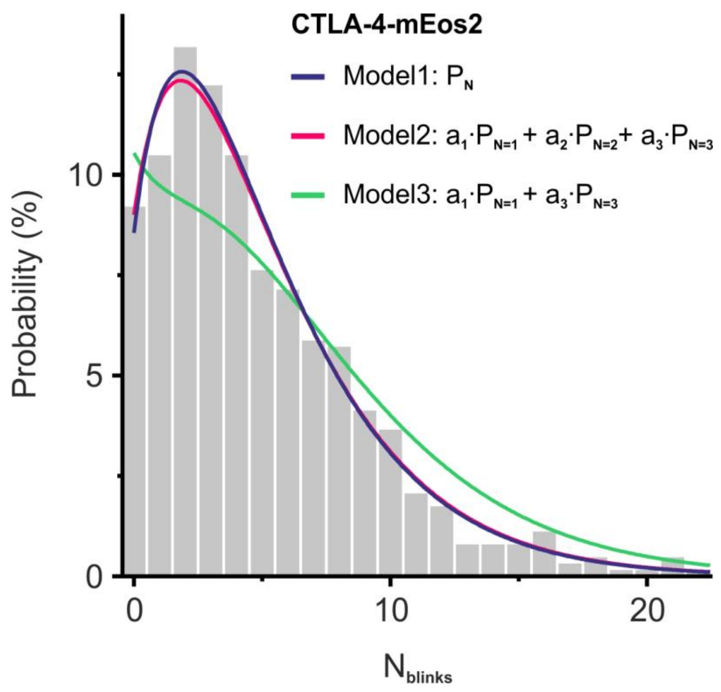

b

Fitting parameter	*Model1*	*Model2*	*Model3*
N	1.93 ± 0.02	NA	NA
a_1	NA	0.06 ± 0.02	0.32 ± 0.03
a_2	NA	0.94 ± 0.07	NA
a_3	NA	0.00 ± 0.05	0.68 ± 0.04
adjusted R^2	0.989	0.989	0.936
AIC	-576.7	-574.8	-484.8

Supplementary Figure S5: Comparison of fitting models for CTLA-4-mEos2. (**a**) The distribution of N_{blinks} is fitted to three different models denoted by Model1 (blue), Model2 (magenta) and Model3 (green). Model1 is a negative binomial distribution with N as fitting parameter. Model2 is a weighted sum of three negative binomial distributions with fixed values of N =1, 2, 3 and weights a_1, a_2, a_3 as fitting parameters respectively. This fit function is employed to evaluate the influence of monomers (N = 1), dimers (N = 2) and trimers (N = 3). Model3 is a weighted sum of two negative binomial distributions with fixed values of N =1, 3 and weights a_1, a_3 as fitting parameters respectively. Model3 is employed to evaluate whether the measured distribution is likely to originate from trimers and monomers only. For all three models, the blink parameter $p^{membrane}$ = 0.28 is kept constant. (**b**) The corresponding fitting parameters, adjusted R^2 and AIC values are listed for all models. The AIC values give decisive conclusion that Model3 is not the correct model, hence CTLA-4 is unlikely to exist as trimers. Model1 and Model2 give similar results, namely that CTLA-4 forms dimers, but no higher-order oligomers. Model2 favors a small monomeric (a_1 = 0.06 ± 0.02) and large dimeric population (a_3 = 0.94 ± 0.07) for CTLA-4, which is

consistent with Model1 with an average number of underlying molecules $N < 2$. This is readily explained by a mEos2 photodetection probability of approximately 90%. Moreover, the AIC test reveals that Model1, which is based on one-parameter fitting and an average number of underlying molecules N, is sufficient to describe the data set.

a

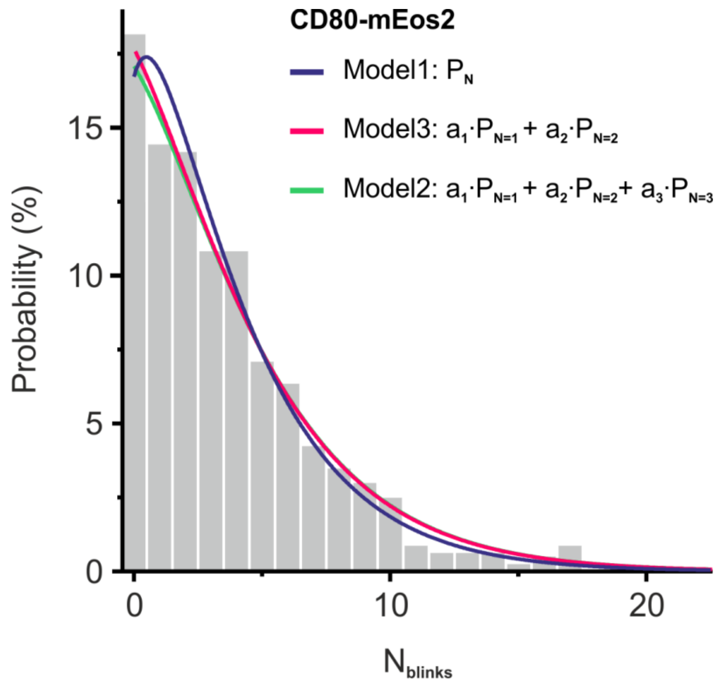

b

Fitting parameter	*Model1*	*Model2*	*Model3*
N	1.41 ± 0.03	NA	NA
a_1	NA	0.48 ± 0.02	0.46 ± 0.03
a_2	NA	0.52 ± 0.03	0.54 ± 0.09
a_3	NA	NA	0.00 ± 0.07
adjusted R^2	0.983	0.990	0.989
AIC	-361.6	-379.5	-375.1

Supplementary Figure S6: Comparison of fitting models for CD80-mEos2. (**a**) The distribution of N_{blinks} is fitted to three different models denoted by Model1 (blue), Model2 (magenta) and Model3 (green). Model1 is a negative binomial distribution with N as fitting parameter. Model2 is a weighted sum of two negative binomial distributions with fixed values of N =1, 2 and weights a_1 and a_2 as fitting parameters respectively. This fit function is employed to evaluate the influence of monomers (N = 1) and dimers (N = 2). In contrast to Model2, Model3 additionally accounts for trimers via a third weighted negative binomial distribution with N = 3 and weight a_3. For all three models, the blink parameter $p^{membrane}$ = 0.28 is kept constant. (**b**) The respective fitting parameters, adjusted R^2 and AIC values are listed. For Model3, no trimeric fraction is obtained (a_3 = 0.00 ± 0.07) supporting our finding that CD80 exists as monomers and dimers but is unlikely to form trimers. We therefore conclude that Model2 is sufficient. To compare Model1 and Model2, we calculated the dimeric fraction for both models taking into account the mEos2 photodetection probability of mEos2 and receive (57 ± 4)% dimers for Model1 and (64 ± 4)%

dimers for Model2. For the latter value, the expected probability $P_{2,2}$ for detecting two functional FPs is accounted for (dimeric fraction = $a_2/P_{2,2}$; for more details see Supplemental Methods).

a

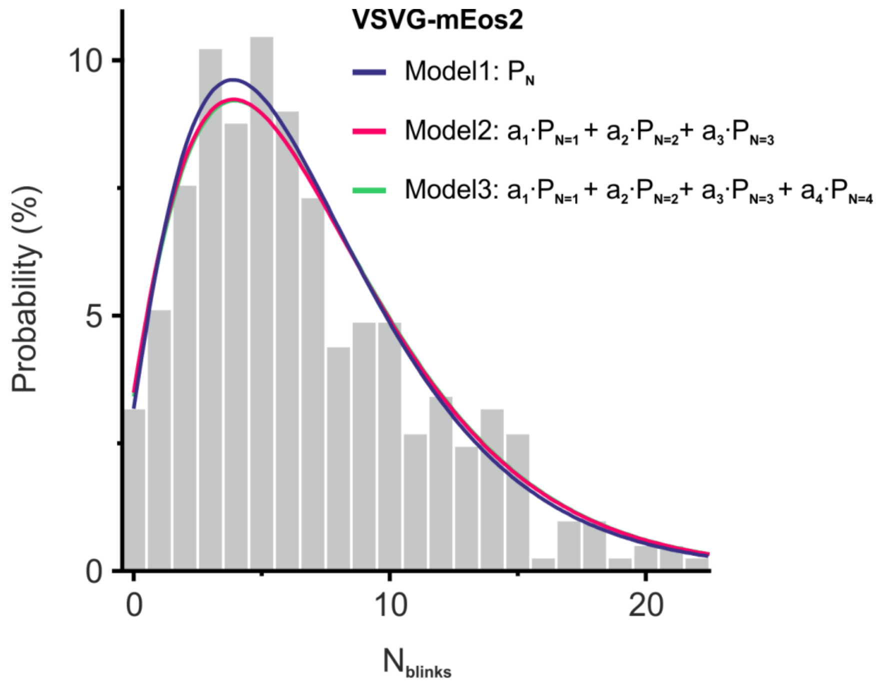

b

Fitting parameter	*Model1*	*Model2*	*Model3*	*Expected probability $P_{n,3}$ for trimer*
N	2.71 ± 0.04	NA	NA	NA
a_1	NA	0.00 ± 0.03	0.00 ± 0.04	0.03 ± 0.01 (n=1)
a_2	NA	0.23 ± 0.08	0.22 ± 0.18	0.24 ± 0.03 (n=2)
a_3	NA	0.77 ± 0.06	0.78 ± 0.28	0.73 ± 0.04 (n=3)
a_4	NA	NA	0.00 ± 0.15	NA
adjusted R^2	0.964	0.961	0.960	NA
AIC	-1139.2	-1129.1	-1126.6	NA

Supplementary Figure S7: Comparison of fitting models for VSVG-mEos2. (**a**) The distribution of N_{blinks} is fitted to three different models denoted by Model1 (blue), Model2 (magenta) and Model3 (green). Model1 is a negative binomial distribution with N as fitting parameter. Model2 is a weighted sum of three negative binomial distributions with fixed values of N =1,2,3 and weights a_1, a_2, a_3 as fitting parameters respectively. This fit function is employed to evaluate the influence of monomers (N = 1), dimers (N = 2) and trimers (N = 3). Model3 additionally accounts for tetramers as it is a weighted sum of four negative binomial distributions with fixed values of N =1,2,3,4 and weights a_1, a_2, a_3, a_4 as fitting parameters respectively. For all three models, the blink parameter $p^{membrane} = 0.28$ is kept constant. (**b**) The obtained fitting parameters, adjusted R^2 and AIC values are listed for all models. The obtained fitting parameters for Model3 indicate that VSVG is unlikely forming tetramers. For evaluation of Model2, we calculated the expected probability $P_{n,3}$ to observe a trimeric complex with three, two and one functional FPs using a

photodetection probability of 90% (see Supplemental Methods). The results are listed in the last column of Fig. S7. The probabilities are in good agreement with the fitting parameter a_1, a_2, a_3 obtained for Model2, suggesting that VSVG is a protein of defined, trimeric stoichiometry at the cell membrane. Note that Model1 also yields trimeric stoichiometry, when the photodetection probability is accounted for, and merely requires a single fitting parameter that is sufficient to describe this data set.

Supplementary Notes

Evaluation of endogenous protein levels

For protein counting using genetic fusion constructs, it is important to be aware of endogenous protein pools that can significantly alter counting results. The viral glycoprotein VSVG has no endogenous expression in Hela cells. To check whether significant amounts of endogenous CD80, CD86 and CTLA-4 receptors were expressed at the surface of the cells and could interfere with mEos2-tagged ones, we stained the receptors by immunofluorescence and performed flow cytometry. Cells were transfected with mEos2 fusion constructs of CD80, CD86 and CTLA-4 respectively under identical conditions as for the SMLM imaging and stained three days later with PE tagged antibody against the respective receptor. As a control, non-transfected Hela cells were incubated with PE tagged antibody against CD80, CD86 and CTLA-4 respectively and their PE signal was compared to cells not incubated with antibody. Two-dimensional dot plots of the flow cytometry data illustrate that cells expressing mEos2-tagged receptors give a clearly visible PE signal, while non-transfected cells showed no visible signal with any of the antibodies. This clearly indicates that the antibodies recognize their respective receptor and, even if some endogenous receptors were expressed, their amount is insignificant compared to the amount of mEos2-tagged receptors at the cell membrane.

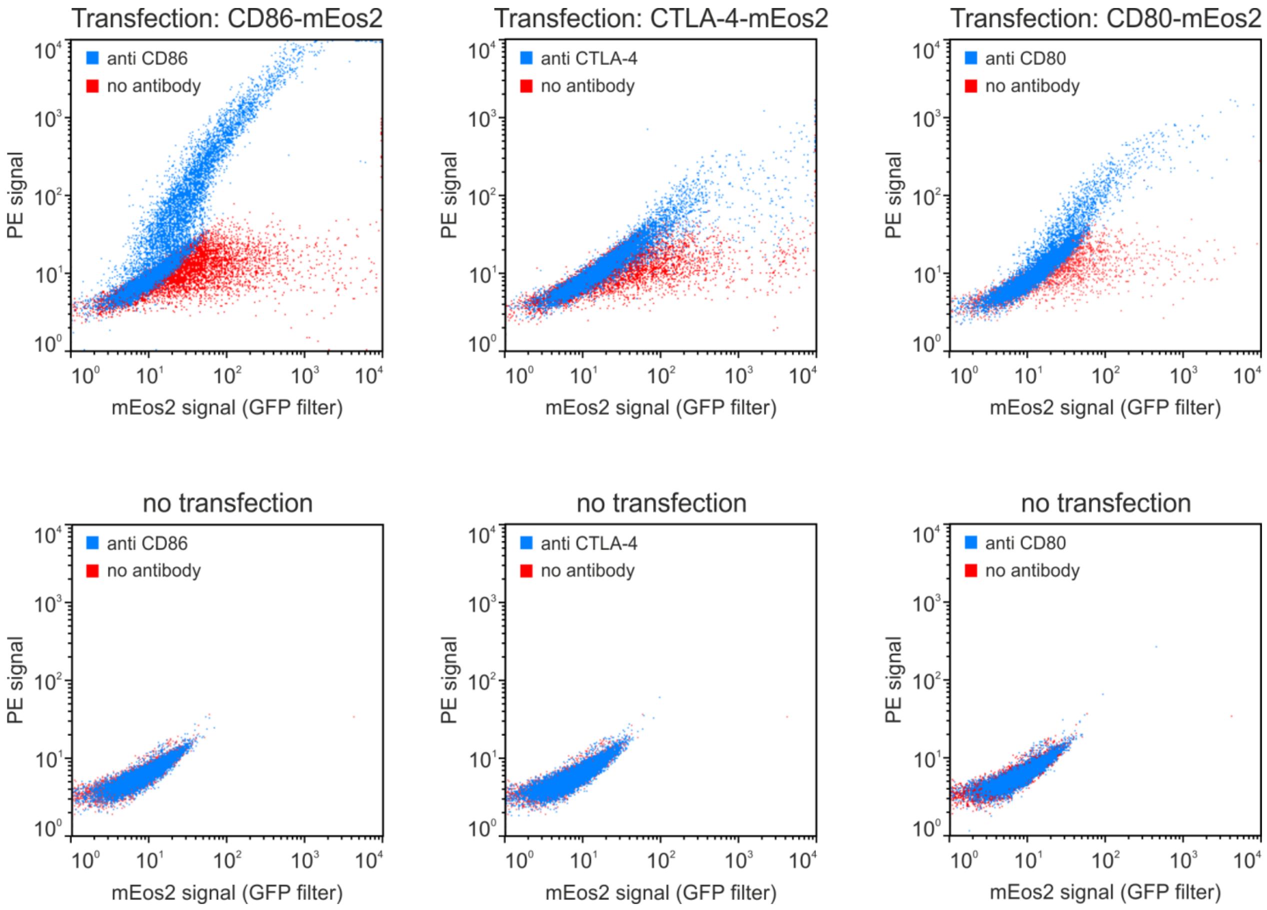

Membrane proteins as calibration standards

We chose monomeric CD86 and covalent dimeric CTLA-4 as calibration proteins, because of their defined stoichiometry at the cell membrane. Both proteins are well-characterized and their stoichiometries validated by several independent studies in the past.

The extracellular part of CTLA-4 receptor consists of a variable immunoglobulin like domain (Ig V) domain. Structural evidence suggests that apo-CTLA-4 exists as a homodimer within the asymmetric unit held together by an extracellular, membrane proximal disulfide bond between two cysteine residues, likely representing the native state[1]. Biochemical[2–4] and resonance energy transfer[5,6] (RET) studies corroborate a purely dimeric assembly.

CD86 is a transmembrane protein with a constant and a variable Ig like domain (Ig C1 and Ig V) on the extracellular side. Several studies using crystallography[7], analytical ultracentrifugation[8], RET[5,6,9] and fluorescence recovery after photobleaching[10] (FRAP) revealed a monomeric assembly for CD86. As a consequence, CD86 is now regularly employed as a monomeric reference for microscopy studies including two recent examples using single-molecule photobleaching / intensity distribution analysis[11] and fluorescence correlation spectroscopy (FCS)[12].

For clarity, crystal structures of the two proteins are depicted below (PDB IDs: 1NCN, 3OSK; visualized with PyMOL).

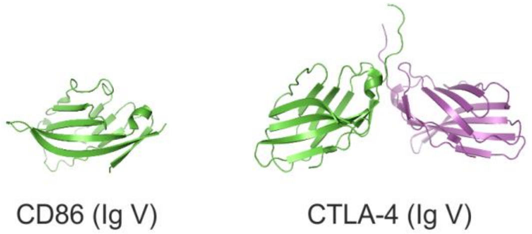

Supplementary Methods

For probing protein stoichiometries, it has to be considered that the photodetection probability s of the fluorescent probe is < 100%. Consequently, a trimer, for example, will appear as dimer or monomer, when not every fluorescent probe is photodetected. The expected probability $P_{n,N}$ to observe a certain oligomeric state n is given by a binomial distribution

$$P_{n,N}(s) = \binom{M}{n} s^n (1-s)^{M-n},$$

where M is the true stoichiometry of the complex. This distribution is majorly dependent on the photodetection probability of the fluorescent probe. As an example, for a trimeric complex, the probability to detect three detected tags is $P_{3,3}(s) = s^3$; for two detected tags $P_{2,3}(s) = 3s^2(1-s)$ and for one detected tag $P_{1,3}(s) = 3s^1(1-s)^2$. The photodetection probability s of mEos2 was calculated as $s = N/2$ using the obtained fitting parameter N from CTLA-4-mEos2 data, since the dimeric stoichiometry of this protein is well defined.

1. Yu, C. *et al.* Rigid-body ligand recognition drives cytotoxic T-lymphocyte antigen 4 (CTLA-4) receptor triggering. *J. Biol. Chem.* **286,** 6685–6696 (2011).

2. Linsley, P. S. *et al.* Binding stoichiometry of the cytotoxic T lymphocyte-associated molecule-4 (CTLA-4): A disulfide-linked homodimer binds two CD86 molecules. *J. Biol. Chem.* **270,** 15417–15424 (1995).

3. Greene, J. L. *et al.* Covalent dimerization of CD28/CTLA-4 and oligomerization of CD80/CD86 regulate T cell costimulatory interactions. *J. Biol. Chem.* **271,** 26762–26771 (1996).

4. Darlington, P. J., Kirchhof, M. G., Criado, G., Sondhi, J. & Madrenas, J. Hierarchical regulation of CTLA-4 dimer-based lattice formation and its biological relevance for T cell inactivation. *J. Immunol.* **175,** 996–1004 (2005).

5. Bhatia, S., Edidin, M., Almo, S. C. & Nathenson, S. G. Different cell surface oligomeric states of B7-1 and B7-2: Implications for signaling. *Proc. Natl. Acad. Sci.* **102,** 15569–15574 (2005).

6. James, J. R., Oliveira, M. I., Carmo, A. M., Iaboni, A. & Davis, S. J. A rigorous experimental framework for detecting protein oligomerization using bioluminescence resonance energy transfer. *Nat. Methods* **3,** 1001–1006 (2006).

7. Zhang, X., Schwartz, J.-C. D., Almo, S. C. & Nathenson, S. G. Crystal structure of the receptor-binding domain of human B7-2: insights into organization and signaling. *Proc. Natl. Acad. Sci.* **100,** 2586–2591 (2003).

8. Collins, A. V *et al.* The Interaction Properties of Costimulatory Molecules Revisited. *Immunity* **17,** 201–210 (2002).

9. Girard, T., Gaucher, D., El-Far, M., Breton, G. & Sékaly, R.-P. CD80 and CD86 IgC domains are important for quaternary structure, receptor binding and co-signaling function. *Immunol. Lett.* **161,** 65–75 (2014).

10. Dorsch, S., Klotz, K.-N., Engelhardt, S., Lohse, M. J. & Bünemann, M. Analysis of receptor oligomerization by FRAP microscopy. *Nat. Methods* **6,** 225–230 (2009).

11. Calebiro, D. *et al.* Single-molecule analysis of fluorescently labeled G-protein-coupled receptors reveals complexes with distinct dynamics and organization. *Proc. Natl. Acad. Sci.* **110,** 743–748 (2013).

12. Herrick-Davis, K., Grinde, E., Cowan, A. & Mazurkiewicz, J. E. Fluorescence Correlation Spectroscopy Analysis of Serotonin, Adrenergic, Muscarinic, and Dopamine Receptor Dimerization: The Oligomer Number Puzzle. *Mol. Pharmacol.* **84,** 630–642 (2013).
